# Digital patient twins for personalized therapeutics and pharmaceutical manufacturing

**DOI:** 10.3389/fdgth.2023.1302338

**Published:** 2024-01-05

**Authors:** Rene-Pascal Fischer, Annika Volpert, Pablo Antonino, Theresa D. Ahrens

**Affiliations:** Fraunhofer Institute for Experimental Software Engineering IESE, Kaiserslautern, Germany

**Keywords:** digital patient twin, personalized therapeutics, personalized medicine, pharmaceutical manufacturing, health ecosystem, digitalization, artificial intelligence, internet of things

## Abstract

Digital twins are virtual models of physical artefacts that may or may not be synchronously connected, and that can be used to simulate their behavior. They are widely used in several domains such as manufacturing and automotive to enable achieving specific quality goals. In the health domain, so-called digital patient twins have been understood as virtual models of patients generated from population data and/or patient data, including, for example, real-time feedback from wearables. Along with the growing impact of data science technologies like artificial intelligence, novel health data ecosystems centered around digital patient twins could be developed. This paves the way for improved health monitoring and facilitation of personalized therapeutics based on management, analysis, and interpretation of medical data via digital patient twins. The utility and feasibility of digital patient twins in routine medical processes are still limited, despite practical endeavors to create digital twins of physiological functions, single organs, or holistic models. Moreover, reliable simulations for the prediction of individual drug responses are still missing. However, these simulations would be one important milestone for truly personalized therapeutics. Another prerequisite for this would be individualized pharmaceutical manufacturing with subsequent obstacles, such as low automation, scalability, and therefore high costs. Additionally, regulatory challenges must be met thus calling for more digitalization in this area. Therefore, this narrative mini-review provides a discussion on the potentials and limitations of digital patient twins, focusing on their potential bridging function for personalized therapeutics and an individualized pharmaceutical manufacturing while also looking at the regulatory impacts.

## 1 Introduction

Digital twins of physical assets have been widely used in digitalization settings in various industry sectors such as automated manufacturing, being a key enabler of the Industry 4.0 wave (1) and to deal with quality assurance also in various sectors (2, 3). In recent years the idea of digital twins for patients in combination with rising data science approaches have been established, being considered a transformation driver to advance healthcare. For example, digital patient twins (DPTs) could provide a technical solution for integrating different health attributes, such as health data from wearables, genetic information, or lab results. Wearables have become increasingly popular in recent years: According to a survey conducted from January to April 2019, around 30% of all adults in the United States are currently using wearables on daily basis (4, 5). As a result, not only is the amount of fitness and wellness data collected increasing, but the potential applications are also growing. For instance, wearables are used for atrial fibrillation detection or for diabetes monitoring (6, 7). Moreover, the increasing availability of health data due to wearables and other medical data sources could enable precision medicine for a broader patient population by providing individual therapies to maximizing efficacy and efficiency while reducing costs for the healthcare system (8). In addition, DPTs could strengthen precision medicine approaches by providing user-friendly access to continuous health monitoring and individualized health advice. As an example, the Swedish Digital Twin Consortium has recognized the potential of DTPs and has already developed a strategy for personalized medication. This strategy states that in the future, a patient's digital twin should first be created. Then, these DPTs should be copied, and each copy should be treated with one or more of thousands of drugs to identify the most effective one to be used subsequently for the patient's real-life treatment (9).

However, DPTs are not yet widely established for routine medical purpose—neither for patients nor for medical experts- and still under development for different use cases (10, 11). Although being especially interesting as facilitator for precision medicine, DPTs as a bridge between health monitoring, personalized therapeutics, and pharmaceutical manufacturing are still not discussed at large. Especially for personalized therapeutics like genetically engineered cell-based therapies, DPTs could be a useful tool for designing these individualized therapies and for communicating these to automated processes in pharmaceutical manufacturing. Hence, this mini-review focuses on this novel function for DPTs at the link of health monitoring, precision medicine, and pharmaceutical manufacturing.

## 2 Background on digital twins

The concept of digital twins was first introduced by Grieves in 2002 (12) as part of a presentation about Product Lifecycle Management (PLM). Though it was not called a “digital twin” at that time, it represented every aspect the current technology also depicts, focusing on the data and information transfer between the physical and digital layers (13). Grieves and Vickers (13) propose multiple definitions and refinements of the twin and its use cases. A digital twin in general is virtual model of physical artefacts that may or may not be synchronously connected, and that can be used to simulate the behavior of the artefact. Based on the findings of Zheng, Yang, and Cheng (14) the digital twins aim at visualizing the data of a specific product with an extremely high degree of accuracy to reality.

The theoretical foundations of digital twins, as mentioned by Tao et al. (15), are split into multiple parts. Focusing on modeling and simulation, data fusion, communication, and services. Each tackles a specific challenge faced by either physical devices or conceptual ideas and allows for the interconnectivity between the physical and digital world as well as the respective environment (13, 15). These generic challenges arise for DPTs as well, but are combined with additional requirements, such as regulatory aspects for drug manufacturing or data sovereignty.

## 3 Monitoring healthcare attributes

In addition to traditional monitoring devices and sensors like conventional electrocardiography systems, wearables are becoming more and more popular. With the help of those, data for health, fitness, and general well-being can be conveniently recorded. Hence, they can ensure continuous and location-independent monitoring of the state of health (16). Commercially available wearables, such as smartwatches or fitness trackers, can monitor various body functions: sleep, body temperature, oxygen saturation, respiratory rate, cardiovascular parameters (including resting pulse, heart rate, electrocardiogram, heart rate variability, and blood pressure), or track user mobility data such as activity duration, step count, stride length, or falls (16). However, it should be noted that the quality of wearables can differ depending on the manufacturer and that they do not have to meet essential safety and performance requirements like medical devices.

Besides wearable sensor-based monitoring devices, other Internet of Things (IoT) monitoring devices exist as well. With IoT technologies new monitoring solutions like remote monitoring can be enabled, allowing for lower costs with the same level of care quality or supporting existing monitoring solutions with additional health attributes or automated analyses (17, 18). Using these technologies multiple solutions for monitoring patient-related health data as mentioned above as well as environmental factors of the hospital like carbon monoxide and carbon dioxide levels have been developed utilizing existing sensors and off-the-shelf components like small single-board computers and open software solutions like Android (19–21). This can go as far as allowing medical professionals to access data remotely or on-site in near real-time while alarming medical staff about abnormalities in data (22, 23).

**Figure 1:**
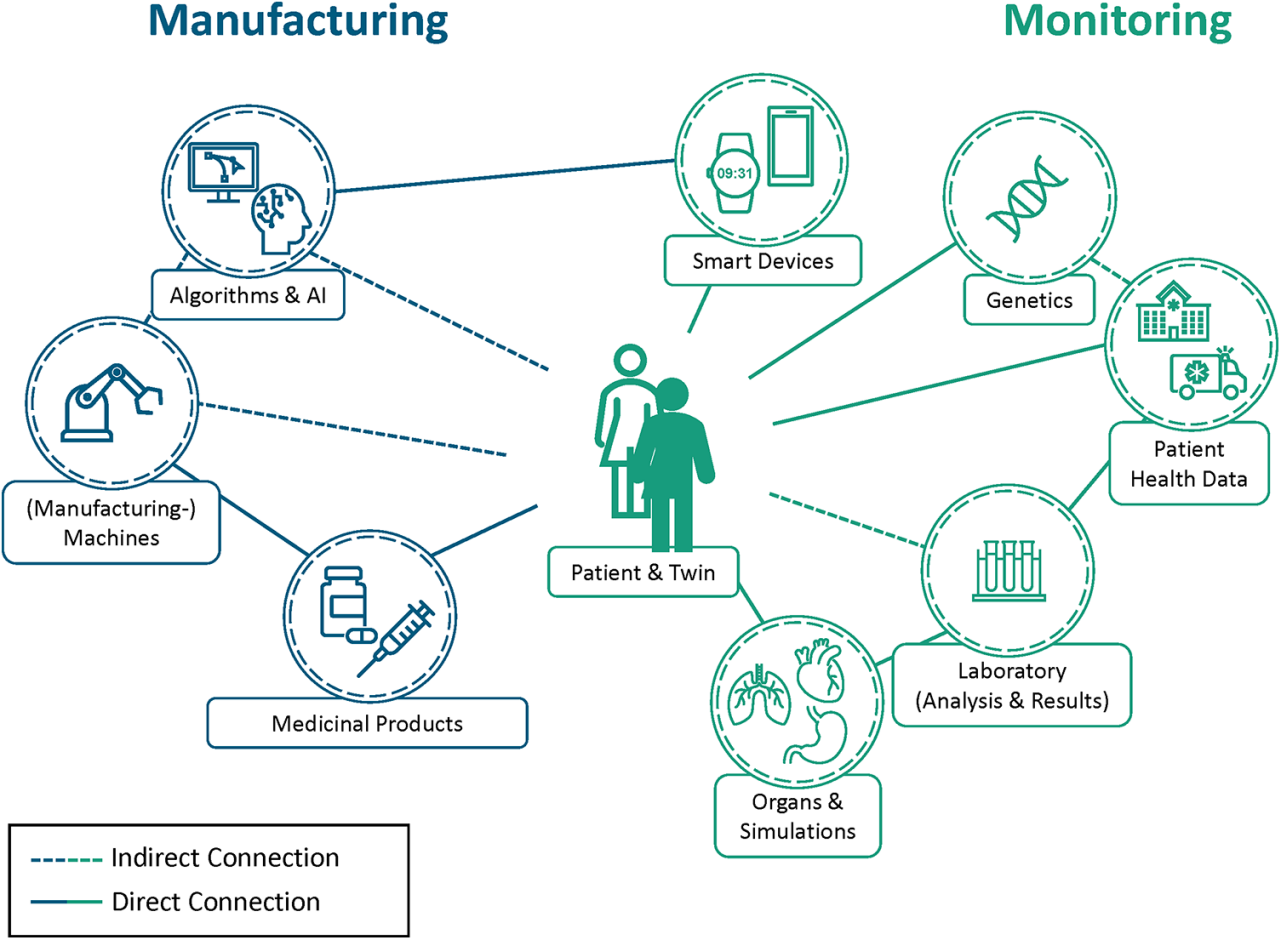
Overview of the digital patient twin (DPT) bridging health monitoring (in green) and pharmaceutical manufacturing (in blue). DPTs (depicted as an filled person icon) contain all information relevant to the patient (highlighted as unfilled person icon) by combining different data sources and analysis results. The gained insights are used to personalize health monitoring, determine potential therapeutics, and improve patient health. As a result, the acquired knowledge can be used in the pharmaceutical manufacturing process to produce individualized therapeutics while digitally monitoring the process and ensuring drug quality and safety. This quality and safety aspect is not only limited to the manufacturing process but can also be used in medical facilities like hospitals or for clinical trials, e.g., by conducting large-scale in silico clinical trials beforehand. This hypothetical health data network gives an overview of the possibilities that DPTs enable, however, it does not show every possible connection between attributes as represented by the connections towards the outside. Highlighted are direct connections between elements like monitoring devices and the patient (shown in solid lines) as well as the indirect connections, as made by the manufacturing devices that rely on data of the DPT to produce the individualized therapeutic (shown in dotted lines). These entities all feed the DPT their own information and use this holistic view to improve themselves. As a result, DPTs can be used for multiple purposes such as the prediction of adverse events or in clinical studies in the future.

As depicted in Figure 1, the DPT (the digital counterpart) corresponds exactly to the physical patient. Some elements, e. g. smart devices like a smartwatch, are interfacing with the person directly, being used for direct monitoring purposes. Others like laboratory research may not be directly linked, but all collected datapoints are documented as part of their respective digital twin and therefore have an indirect connection to the DPT that may reuse the data. Concepts like the DPT aim to unify the aspects of monitoring, artificial intelligence models, and simulations into a single digital asset that enables better interconnectivity and interoperability between sensors and systems, similar to the digital twin concept in Industry 4.0 (24).

## 4 Implications for health monitoring and personalized therapeutics in the future

DPTs have the potential to transform healthcare by improving and potentially accelerating diagnosis and treatment on the one hand, and by mapping clinical pathways or patient care processes on the other hand (11, 25). Despite these opportunities, experts note that there is still a lack of user-friendly software for patients and physicians to use digital twins (25, 26).

Currently, digital twins in the healthcare sector are mostly developed in research settings, spanning digital twins to represent the entire human body, a body system or function, an organ, a specific cell type, or even a specific subcellular or molecular level within a cell. Examples of digital twins of organs include the heart for the development of safe and effective cardiovascular products or the brain for targeted epilepsy surgery (27, 28). Furthermore, the digital twins can also be used for holographic systems. For instance, Gong et al. (29) are describing an interactive platform for visualizing the digital twin of a human heart created by computed tomography images. The integration of digital twins into the metaverse is proposed by Wang et al. (30) to promote smart healthcare. As an example, this would allow digital twins of computer tomography scanners to examine digital patient avatars.

Further, DPTs can also focus on specific diseases. Voigt et al. (31) are investigating digital twins for individualized disease management and optimized treatment of multiple sclerosis. In addition, the digital twin enables the representation of the clinical pathway of the patient. Therefore, its attributes are based on various characteristics of multiple sclerosis, such as parameters from neurological examinations, functional tests, imaging, neurobiological and immunological data, and information about the patient's life circumstances (31). The European DIGIPREDICT project is researching a digital twin to represent patient-specific physiology and predict the course of viral infection diseases with the help of smart patches. Among other things, DIGIPREDICT aims to forecast the severity of COVID 19 symptoms. The main goal of the project is to enable the identification, monitoring, and screening of high-risk patients and thus suggesting the right (supportive) therapy (32).

Since DPTs integrate various data sources and aid clinical interpretation and decision making, they are becoming an integral part of precision medicine to select the most effective drug for individual patients. With this, DPTs could be used for management and monitoring of chronic diseases. Diabetes Type 2 is a widespread chronic disease with potential long-term organ damage due to increased blood glucose levels, with consequent dramatic loss of quality of life and immense socio-financial burden. As a result, the management of diabetes with DPTs is currently being discussed (33). For instance, DPTs are giving individualized advice on nutrition, leading to better controlled blood glucose levels. Indeed, DPTs have been already evaluated in clinical studies for diabetic patients (34). In one of those studies machine learning algorithms were applied to predict the glycemic responses to food. Based on these results, individualized recommendations were given to reduce blood glucose spikes (34). DPTs can also guide treatment decisions based on outcome prediction. For example, Venkatapurapu et al. (35) have evaluated a computational approach to predict temporal changes in mucosal health in Crohn's disease. With this approach, they aim to support physicians' decisions when considering the state of gastrointestinal tissue in patients.

Another interesting use case is the reliable prediction of drug efficacy and side effects for patients before drug administration, which is currently not fully achievable for clinicians by conventional methods. Prediction and detection of drug adverse events is currently under development in research settings (36, 37). Although clinical trials are used to assess the effectiveness and safety of new drugs, it is hardly possible to detect all side effects and drug interactions in clinical trials, as the number of participants is not enough to catch every possible side effect. As a result, adverse drug events are a considerable risk for mortality and increased costs in hospitalized patients (38).

DPTs could help to predict these side effects prior to the actual treatment. The clinical trial of Swen et al. (39) showed that genetic information can be used to reduce adverse drug reactions in patients, paving the way for routine pharmacogenetic-based prediction of drug adverse events in the future. Therefore, it is suggested that DPTs created using genetic information can be used during personalized treatments or to evaluate new drugs (40). Individualized therapeutics are especially progressing in cancer therapies, e.g., one approach is the identification of so called neoantigens and subsequent individualized vaccination for tumor immune therapy (41). Clinical studies show that this strategy is clinically effective in several cancer entities such as melanoma (42) or prostate cancer (43). However, mass production of such individualized vaccines and other Advanced Therapy Medicinal Products (44) is not yet established and still challenging. DPTs could guide the design of such individualized therapeutics and allow automated pharmaceutical manufacturing at the same time.

## 5 Digital patient twins interfacing pharmaceutical manufacturing and regulatory aspects

Digital Twins are already being used to optimize the manufacturing process in the pharmaceutical industry by improving long production processes and long medical release times and thus increasing efficiency (9, 45) or machine learning and artificial intelligence techniques in the drug discovery and development process (46, 47). Figure 1 shows how pharmaceutical manufacturing can be connected to the DPT. While the drugs in the figure are a directly linked to the DPT, manufacturing equipment is not; it is only indirectly connected to the DPT via the drugs relying on the data the DPT provides to adjust to the requirement. Nevertheless, the concept of personalized therapeutics, where patient data is incorporated into the manufacturing process to develop the most effective therapeutic, is still influenced by this missing connection.

Since every device used in manufacturing and in medical settings in general, including software, requires regulatory approval, novel approaches will face hurdles that need to be overcome. However, DPTs have, at least in part, been approved in the past by the European Medicines Agency for certain predictions in Phase 2 and 3 clinical trials (48). Simulation models for physiologically based pharmacokinetics have their own guidelines presented by major agencies such as the U.S. Food and Drug Administration (FDA) (49) or European Medicines Agency (EMA) (50) as their usage has increased and their usefulness has been proven (51, 52).

Regulatory agencies like the EMA and FDA recommend ways to incorporate new digital solutions in the development/production of new drugs in general (53–55), but do not provide specific guidelines for these concepts. This is especially prevalent for individualized drugs with the goal of lot size one, as every therapeutic potentially needs its own regulatory approval. As a result, the pharmaceutical domain calls for more specifications in digital health technologies to have a more streamlined approval process (53, 56).

## 6 Conclusion

The digital transformation has fundamentally changed many aspects of our daily life. However, the digitization of healthcare still lags behind other sectors. The evolving concepts around DPTs, along with data science and artificial intelligence approaches like machine learning are paving the way for novel healthcare monitoring. DPTs can be a user-friendly and easily accessible tool to increase health data literacy in the population. In combination with reliable and robust artificial intelligence algorithms for health surveillance and prediction of health risks, health management could be shifted from disease treatment to prevention. As DPTs are integrating sensitive health data, strict systems for data control and data security are needed. Interestingly, digital twins have already been proposed as an integral part of cybersecurity for healthcare applications (57) as well as manufacturing (58). Moreover, similar to other domains, health data sovereignty should be in focus to increase the trust in these new solutions to achieve the targeted goals (59). Moreover, ethical concerns regarding prediction of disease onset of untreatable diseases and other potential challenges within digital health need to be carefully considered in the future and before implementation. However, a detailed discussion of these aspects is beyond the scope of this review although being highly relevant.

Overall, DPTs are especially interesting for precision medicine and personalized therapeutics as access to individual health information is rapidly increasing. One example is the bigger access to genetic information which is paving the way for individualized prediction of drug adverse events as shown in the clinical study by Swen and colleagues (39). This is important due to high costs for innovative therapies which can be lowered using fully automated pharmaceutical manufacturing systems while improving sustainability with simulations of the efficacy and overall usefulness of the proposed therapy. Ecosystems centered around DPTs for health monitoring, design of personalized therapeutics, and prediction of drug efficacy have enormous potential for restructuring health care systems. Therefore, DPTs could be a bridging tool to pharmaceutical manufacturing of individualized therapeutics as long as regulatory aspects and quality control systems are adopted to it.
